# *DEPDC5* Variants Associated Malformations of Cortical Development and Focal Epilepsy With Febrile Seizure Plus/Febrile Seizures: The Role of Molecular Sub-Regional Effect

**DOI:** 10.3389/fnins.2020.00821

**Published:** 2020-08-11

**Authors:** Liu Liu, Zi-Rong Chen, Hai-Qing Xu, De-Tian Liu, Yong Mao, Han-Kui Liu, Xiao-Rong Liu, Peng Zhou, Si-Mei Lin, Bin Li, Na He, Tao Su, Qiong-Xiang Zhai, Heng Meng, Wei-Ping Liao, Yong-Hong Yi

**Affiliations:** ^1^Institute of Neuroscience, Department of Neurology of the Second Affiliated Hospital of Guangzhou Medical University, Key Laboratory of Neurogenetics and Channelopathies of Guangdong Province and the Ministry of Education of China, Guangzhou, China; ^2^Department of Neurology, Xiaoshan First People’s Hospital, Hangzhou, China; ^3^Department of Neurology, The First Affiliated Hospital of Guangxi Medical University, Nanning, China; ^4^Department of Neurology, Xuzhou Central Hospital, Affiliated Hospital of Southeast University, Xuzhou, China; ^5^BGI-Shenzhen, Shenzhen, China; ^6^Department of Pediatrics, Guangdong General Hospital, Guangdong Academy of Medical Sciences, Guangzhou, China; ^7^Department of Neurology of the First Affiliated Hospital of Jinan University and Clinical Neuroscience Institute of Jinan University, Guangzhou, China

**Keywords:** *DEPDC5*, focal epilepsy, febrile seizures, genotype–phenotype correlation, molecular sub-regional effect

## Abstract

To explore the phenotype spectrum of *DEPDC5* variants and the possible mechanisms underlying phenotypical variation, we performed targeted next-generation sequencing in 305 patients with focal epilepsies and 91 patients with generalized epilepsies. Protein modeling was performed to predict the effects of missense mutations. All previously reported epilepsy-related *DEPDC5* variants were reviewed. The genotype–phenotype correlations with molecular sub-regional implications were analyzed. We identified a homozygous *DEPDC5* mutation (p.Pro1031His) in a case with focal cortical dysplasia and eight heterozygous mutations in 11 families with mild focal epilepsies, including 13 patients in eight families with focal epilepsy with febrile seizures plus/febrile seizures (FEFS + /FS). The mutations included one termination codon mutation (p.Ser1601_Ter1604del_ext133), three truncating mutations (p.Val151Serfs^∗^27, p.Arg239^∗^, and p.Arg838^∗^), and four missense mutations (p.Tyr7Cys, p.Tyr836Cys, p.Pro1031His, and p.Gly1545Ser) that were predicted to affect hydrogen bonds and protein stability. Analysis on epilepsy-related *DEPDC5* variants revealed that malformations of cortical development (MCDs) had a tendency of higher frequency of null mutations than those without MCD. MCD-associated heterozygous missense mutations were clustered in structural axis for binding arrangement (SABA) domain and close to the binding sites to NPRL2/NPRL3 complex, whereas those associated with FEFS + /FS were a distance away from the binding sites. Evidence from four aspects and one possible evidence from sub-regional implication suggested MCD and FEFS + /FS as phenotypes of *DEPDC5* variants. This study suggested that the phenotypes of *DEPDC5* variants vary from mild FEFS + /FS to severe MCD. Heterozygous *DEPDC5* mutations are generally less pathogenic and commonly associated with mild phenotypes. Bi-allelic mutations and second hit of somatic mutations, together with the genotype–phenotype correlation and sub-regional implication of *DEPDC5* variants, explain severe phenotypes.

## Introduction

*DEPDC5* gene (OMIM^∗^614191) encodes disheveled Egl-10 and pleckstrin (DEP) domain containing protein 5, which forms part of the GTPase-activating protein activity toward RAG complex 1, a repressor of the mammalian target of rapamycin (mTOR) signaling pathway that is closely related to epilepsies (Bar-Peled et al., 2013; Baldassari et al., 2019). The mTOR pathway is involved in a myriad of biological processes, including cell growth, proliferation, and protein synthesis (Fingar and Blenis, 2004; Sarbassov et al., 2005; Marsan and Baulac, 2018). *DEPDC5* mutations have been demonstrated to be the most common cause of familial focal epilepsies (Dibbens et al., 2013). However, the phenotypes of *DEPDC5* mutations included not only familial epilepsies such as familial focal epilepsy with variable foci (Dibbens et al., 2013), autosomal dominant (AD) nocturnal frontal epilepsy (Ishida et al., 2013; Picard et al., 2014), and familial temporal lobe epilepsy (TLE) (Ishida et al., 2013; Striano et al., 2015) but also non-familial focal epilepsies, such as childhood epilepsy with centrotemporal spikes (rolandic epilepsy) (Lal et al., 2014) and epilepsy with continuous spikes and waves during slow-wave sleep (Ricos et al., 2016). In addition, cases of focal cortical dysplasia (FCD) (Scerri et al., 2015) and hemimegalencephaly (D’Gama et al., 2015) were reported. On the other hand, *DEPDC5* mutations have been occasionally identified in cases of epileptic spasms (Carvill et al., 2015); asymptomatic carriers were also common across the *DEPDC5* mutation-related families (Tsai et al., 2017). Therefore, the phenotypic spectrum of *DEPDC5* mutations requires further verification, and it is unknown whether the phenotypic variation is correlated with the genotypes of *DEPDC5* mutations.

Here, we performed targeted next-generation sequencing approach in a cohort of patients with focal epilepsies or generalized epilepsies. Eight *DEPDC5* variants were identified in 12 unrelated families with phenotypic heterogeneity, including eight families with 13 individuals with focal epilepsy with febrile seizures plus/febrile seizures (FEFS + /FS) and a homozygous mutation in a case with FCD. We systematically reviewed the *DEPDC5* variants and analyzed the genotype–phenotype correlation, with special attention on the molecular sub-regional implications of mutations, which was suggested to be a critical consideration in evaluating the pathogenicity of sequence variants (Gussow et al., 2016; Tang et al., 2019). To determine the association between *DEPDC5* variants and malformations of cortical development (MCDs) or FEFS + /FS, evidence from five clinical-genetic aspects was analyzed.

## Materials and Methods

### Patients

We recruited a cohort of patients with epilepsies, which consisted of 305 patients with focal epilepsies and 91 patients with generalized epilepsies. The patients were from the Epilepsy Center of the Second Affiliated Hospital of Guangzhou Medical University and Guangdong General Hospital from July 2015 to November 2019. Patients with focal epilepsies included 20 cases with FCD, considering that mutations of several genes were associated with focal epilepsies and cortical malformation (Guerrini, 2005). Patients with focal epilepsies caused by acquired etiologies, such as tumor, trauma, and stroke, were excluded. All individuals enrolled were unrelated ethnic Han Chinese who lived in southern China. None of the biological grandparents of the participants were from other races.

The collected clinical data included seizure onset age, seizure type and frequency, response to antiepileptic drugs, general and neurological examination results, and detailed family history. Magnetic resonance imaging (MRI) scans were performed to detect any brain structure abnormalities on a 3.0-T Magnet including three-dimensional T1 and T2 weighted, T2 fluid-attenuation inversion recovery (FLAIR) in a horizontal position, T1 weighted and T2 FLAIR in a coronary position, T2 weighted in a sagittal position, 3.0-mm-thick contiguous slices, and sequences. Long-term (24-h) video electroencephalography (EEG) monitoring records that included hyperventilation, intermittent photic stimulation, open–close eyes test, and sleeping recording were obtained. Epileptic seizures and epilepsies were diagnosed according to the criteria of the Commission on Classification and Terminology of the International League Against Epilepsy (ILAE) (1981, 1989, 2001, 2010, and 2017). Focal epilepsies were diagnosed based on focally originated seizures and/or focal discharges of EEG. Generalized epilepsies were diagnosed based on a range of seizure types including absence, spasms, myoclonic, clonic, atonic, tonic, and tonic–clonic seizures, supported by the finding of typically generalized discharges on EEG.

This study adhered to the guidelines of the International Committee of Medical Journal Editors regarding patient consent for research or participation. Written informed consent was obtained from the individuals or legal guardians. This study was approved by institutional review board and ethics committee of the Second Affiliated Hospital of Guangzhou Medical University (approval number: 2014004).

### Targeted Sequencing

Whole blood samples were collected from the probands, their parents, and other available relatives to ascertain if the variants were inherited or *de novo* and for cosegregation analysis. Genomic DNA was extracted from whole blood using the Qiagen Flexi Gene DNA Kit (Qiagen, Hilden, Germany).

A gene panel was designed for targeted sequencing of 483 genes that are possibly associated with epilepsy to uncover disease-causing variants (Supplementary Table S1) (Zhou et al., 2018). Genes potentially associated with focal epilepsies in the panel included *CHRNA2*, *CHRNA4*, *CHRNB2*, *CNTNAP2*, *DEPDC5*, *FLNA*, *GABRG2*, *GRIN2A*, *KCNQ2*, *KCNQ3*, *KCNT1*, *LGI1*, *MECP2*, *NPRL2*, *NPRL3*, *PCDH19*, *POLG*, *PRIMA1*, *PRRT2*, *RELN*, *SCN1A*, *SCN1B*, *SCN2A*, *SLC2A1*, *SRPX2*, *SYN1*, *TBC1D24*, *TSC1*, and *TSC2*. The sequencing method and filtering criteria were as those described previously (Zhou et al., 2018). All candidate pathogenic variants were validated by Sanger sequencing. Paternity and maternity of the probands were confirmed by alignment of the segregated sequence variants.

### Molecular Structural Analysis

Protein modeling was performed to predict the effects of missense variants on molecular structure by using Iterative Threading ASSEmbly Refinement (I-TASSER) software (Yang et al., 2015), based on the updated template of 6CES.pdb (chain D)^1^ (Shen et al., 2018). PyMOL 1.7 was used for three-dimensional protein structure visualization and analysis. We used mCSM to predict protein stability, which is indicated by free energy change (ΔΔ*G*) (Pires et al., 2014). Mutations were discriminated into two classes: destabilizing mutations (ΔΔ*G* < 0 kcal/mol) and stabilizing mutations (ΔΔ*G* > 0 kcal/mol). Free SASA is used to calculate solvent-accessible surface area (SASA) (Mitternacht, 2016).

### Genotype–Phenotype Correlation

We retrieved all *DEPDC5* mutations from the PubMed^2^ and the HGMD^3^ up to November 2018 (Supplementary Table S2). All *DEPDC5* mutations were validated by direct sequencing in the original reports. We rechecked all the mutations with nucleotide and amino acid numbering according to *DEPDC5* reference transcript NM_001242896.1 (reference protein NP_001229825.1). To avoid duplicate recruitment, mutations were cross-referenced on their genetic and clinical information.

To facilitate analyzing the correlation between genotype and phenotype, gene mutations are classified into null and missense mutations. Null mutations are those causing gross protein malformations, including truncating mutations (nonsense and frameshifting), splice-site mutations, and mutations at initiation codon or with single/multiple exon deletion, which mainly lead to complete loss of function and haploinsufficiency (Richards et al., 2015).

Phenotypes of the *DEPDC5* mutations were listed according to the original reports. Familial case was defined by existence of at least two members carrying the same *DEPDC5* mutation. Families with only one affected individual (due to incomplete penetrance) were indicated. An affected family was considered a single case in data analysis. For our analysis, cases with MCD were separated from those without MCD. A familial case of MCD was defined by existence of at least one affected member presented MCD; and the detailed number of affected members with MCD or other phenotypes was indicated (Supplementary Table S2). The cases without MCD were further classified into cases with focal epilepsy and cases with FEFS + /FS. FS + was used to denote the individuals with FS extending outside the age range of 3 months to 6 years, or with afebrile seizures. It was observed in several mutations that the same mutation was reported to be associated with different phenotypes (Supplementary Table S2). The penetrance of *DEPDC5* mutation was defined as the proportion of affected individuals with the mutation, that is, the number of affected individuals with the mutation divided by the total number of individuals with the mutation. A family, or sub-branches within a family, was recruited for penetrance analysis when all individuals in the family or sub-branches of the family were tested for *DEPDC5* mutations (Meng et al., 2015).

### Evaluating a Phenotype of *DEPDC5* Variants

To determine the association between *DEPDC5* variants and MCD or FEFS + /FS, evidence from five clinical-genetic aspects was analyzed. These include (1) whether variants were recurrently identified in unrelated cases of homogenous phenotype, or significantly high frequency, or hotspot in patient group (repetition); (2) for heterogeneous phenotypes, whether a phenotype was within a spectrum that was correlated with genotype (genotype–phenotype correlation); (3) defined inheritance pattern, for example, cosegregation in families with AD/autosomal recessive (AR) inheritance pattern, or mainly *de novo* origination (inheritance pattern); (4) correlation between genetic impairment and phenotype severity (genetic quantitative correlation); and (5) defined sub-regional (functional domains) or sub-molecular implications of the variants (molecular sub-regional implication), or distinct pathogenic functional alteration/mechanism.

### Statistical Analysis

Statistical analyses were performed with the SPSS version 23.0 (SPSS, Inc., Chicago, IL, United States). Chi-square test or Fisher’s exact test was applied to compare the frequencies of null/*de novo* mutations and penetrance between different genotype groups. The relationship between phenotype and the occurrence of null/*de novo* mutations was analyzed by Spearman’s correlation test. Values of *p* < 0.05 (two-sided) were considered significant.

## Results

### Identification of Novel *DEPDC5* Variants

**FIGURE 1:**
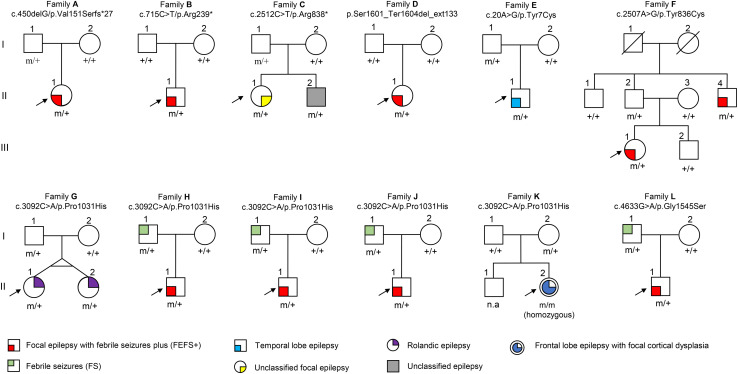
Pedigrees of the families with *DEPDC5* mutations. Individuals with heterozygous mutation are indicated by m/+, those with homozygous mutation are indicated by m/m, and those negative for mutation are indicated by +/+. The probands are indicated by arrows. n.a., not available. The phenotype of each case is indicated by different symbols in the figure.

Among the 305 patients with focal epilepsies, eight *DEPDC5* mutations were identified in 12 unrelated families (Figure 1 and Table 1, sequencing graph, see Supplementary Figure S1). A heterozygous truncating mutations (p.Val151Serfs^∗^27), a termination codon mutation (p.Ser1601_Ter1604del_ext133), and two missense mutations (p.Tyr836Cys and p.Gly1545Ser) have not been reported previously and were novel findings. Truncating mutation p.Arg239^∗^ has been reported in cases of FCD and focal epilepsy (Ishida et al., 2013; Baulac et al., 2015; Baldassari et al., 2019), whereas mutation p.Arg838^∗^ has been identified in cases of sleep-related hypermotor epilepsy, frontal lobe epilepsy (FLE), or focal epilepsy (Baldassari et al., 2019). Mutation p.Tyr7Cys has previously been reported in a case of TLE (Tsai et al., 2017). Mutation p.Pro1031His, which has been described previously as a *de novo* mutation in a patient with late-onset epileptic spasms with focal discharges (Carvill et al., 2015), appeared as a heterozygous variant in four families with FEFS + /FS or rolandic epilepsy and as homozygous variant in a case of FLE with FCD. In contrast, no *DEPDC5* mutation was identified in any of the 91 patients with generalized epilepsies.

**TABLE 1:** Clinical feature of patients with *DEPDC5* mutations.

Patient	Mutation	Current age	Gender	FS	aFS^a^	Onset age	Frequency of seizure	EEG	MRI	Phenotype	AEDs	Outcome
**A: II-1**	p.Val151Serfs^∗^27	24 years	Female	GTCS	SPS	4 years	3–4/day	FSW	Normal	FS +	LEV	Sz free
**B: II-1**	p.Arg239^∗^	17 years	Male	GTCS	GTCS	15 years	4/year	SW, SSW	NA	FS +	LEV	Sz free
**C: II-1**	p.Arg838^∗^	31 years	Female	-	**sGTCS**,	7 years	1–2/month	GPSW, Multi. FD	Normal	UFE	VPA	Sz free
**C: II-2**	p.Arg838^∗^	28 years	Male	-	sGTCS	16 years	3 epis.	NA	NA	UE	-	Sz free
**D: II-1**	p.Ser1601_Ter 1604del_ext133	6 years	Female	CPS	CPS	3.8 years	3–5/years	FSSW	NA	FS +	LEV	Sz free
**E: II-1**	p.Tyr7Cys	12 years	Male	-	sGTCS	11 years	2 epis.	FSW	L.hip.atrop	TLE	OXC	Sz free
**F: III-1**	p.Tyr836Cys	22 years	Female	GTCS	sGTCS	2 years	4–6/year	FSW	Normal	FS +	VPA, LEV	Improved
**F: II-4**	p.Tyr836Cys	58 years	Male	GTCS	sGTCS	2 years	4–6/year	NA	NA	FS +	NA	Sz free
**G: II-1**	p.Pro1031His	6 years	Female	-	**sGTCS**, CPS	5 years	1/month	Multi. FD	Normal	RE	LTG, VPA	Improved
**G: II-2**	p.Pro1031His	6 years	Female	-	sGTCS	6 years	2 epis.	Multi. FD	Normal	RE	ICM	Sz free
**H: II-1**	p.Pro1031His	6 years	Male	GTCS	sGTCS	1.5 years	2–3/year	GSW, Multi. FD	Normal	FS +	VPA	Improved
**H: I-1**	p.Pro1031His	31 years	Male	GTCS	-	2 years	1 epis.	NA	NA	FS	NA	Sz free
**I: II-1**	p.Pro1031His	8 years	Male	GTCS	sGTCS	8 months	1/year	FSW	Normal	FS +	TPM, OXC	Sz free
**I: I-1**	p.Pro1031His	40 years	Male	GTCS	-	3 years	2 epis.	NA	NA	FS	-	Sz free
**J: II-1**	p.Pro1031His	7 years	Female	GTCS	sGTCS	2 years	2/year	FSW	Normal	FS +	LTG	Improved
**J: I-1**	p.Pro1031His	39 years	Male	GTCS	-	2 years	1/year	NA	NA	FS	-	Sz free
**K: II-2**	p.Pro1031His (homozygous)	5 years	Female	CPS	-	3 years	7–8/day	FSW	Abnormal	FLE with FCD	LEV, OXC, CNZ	Sz free
**L: II-1**	p.Gly1545Ser	7 years	Male	sGTCS	-	3 years	2 epis	Normal	Normal	FS +	-	Sz free

*Abbreviations: AEDs, antiepileptic drugs; aFS, afebrile seizures; CNZ, clonazepam; CPS, complex partial seizure; epis, episode; FLE, frontal lobe epilepsy; FS, febrile seizures; FSW, focal spikes and waves; FS +, febrile seizures plus; GPSW, generalized polyspikes and slow waves; GSW, generalized spikes and waves; GTCS, generalized tonic–clonic seizures; ICM, ilepcimide; LEV, levetiracetam; LTG, lamotrigine; MCD, malformations of cortical development; Multi. FD, multifocal discharges; NA, not available; OXC, oxcarbazepine; RE, rolandic epilepsy; sGTCS, secondary generalized tonic–clonic seizures; SPS, simple partial seizure; SSW, spikes and slow waves; SW, sharp waves; FSSW, focal sharp slow waves; TLE, temporal lobe epilepsy; TPM, topiramate; UE, unclassified epilepsy; UFE, unclassified focal epilepsy; VPA, valproate. ^*a*^In bold are the main seizure types, with seizure frequency indicated in the following column.*

**FIGURE 2:**
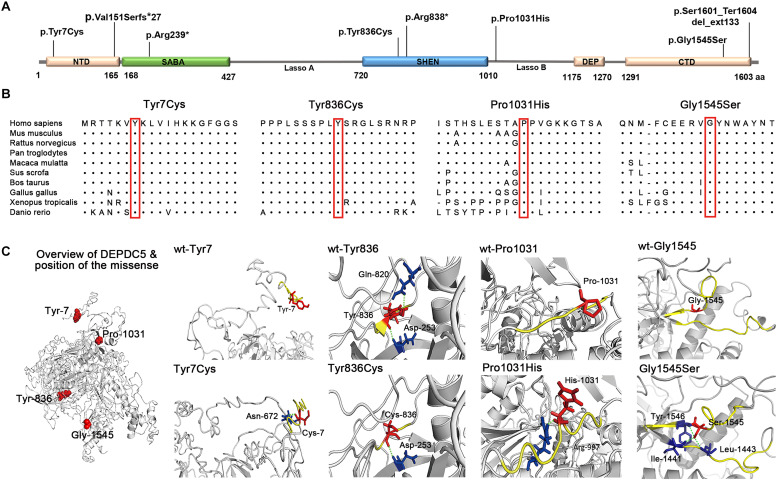
Schematic presentation of DEPDC5 structure. **(A)** The positions of the mutations identified in this study are indicated relative to functional domains. **(B)** All four missense mutations identified in this study affected amino acid residues that are highly conserved in various species. **(C)** Overview of DEPDC5 and focal structures of wild type and mutants. The residues where the mutations occurred are shown as red rods. The hydrogen-bonded residues are colored blue. The hydrogen bonds are shown as green spheres. The yellow-colored structure where Tyr836 located is a short β sheet. NTD, N-terminal domain; SABA, structural axis for binding arrangement; SHEN, steric hindrance for enhancement of nucleotidase activity; DEP, disheveled, Egl-10 and pleckstrin; CTD, C-terminal domain.

We analyzed the potential molecular effects of the variants. A recent study demonstrated that DEPDC5 contains five functional domains, including N-terminal domain (NTD), structural axis for binding arrangement (SABA) domain, steric hindrance for enhancement of nucleotidase activity (SHEN) domain, DEP domain, and C-terminal domain (CTD) (Shen et al., 2018). Locations of the eight mutations identified in this study are indicated in Figure 2A.

Truncating mutations (p.Val151Serfs^∗^27, p.Arg239^∗^, and p.Arg838^∗^) and termination codon mutation (p.Ser1601_Ter1604del_ext133) were not present in ExAC, 1000 Genomes, or gnomAD database. They could cause gross protein malformations and lead to functional haploinsufficiency. The four missense mutations substituted evolutionarily conserved amino acid residues (Figure 2B). These missense mutations presented in ExAC, 1000 Genomes, and gnomAD databases as a minor allele frequency of < 0.005 and were suggested to be damaging or possibly damaging by the web-based prediction tools (SIFT, PolyPhen-2, and Mutation Taster) (Table 2). However, p.Tyr7Cys, p.Tyr836Cys, and p.Pro1031His present at higher frequencies in East Asian population than that in general populations in ExAC database (0.0007 vs. 0.00005, 0.0013 vs. 0.0001, and 0.0076 vs. 0.0005, respectively). With the use of the standards and guidelines for the interpretation of sequence variants by the American College of Medical Genetics and Genomics, evaluation of pathogenicity of the variants showed that p.Arg239^∗^ and p.Ser1601_Ter1604del_ext133 were pathogenic; p.Arg838^∗^, p.Val151Serfs^∗^27, and p.Pro1031His were likely pathogenic; and p.Tyr7Cys, p.Tyr836Cys, and p.Gly1545Ser were of uncertain significance (Table 2).

**TABLE 2:** *In silico* analysis and ACMG scoring of *DEPDC5* variants.

Mutation	ExAC-All (East Asian)	KG-All (East Asian)	gnomAD (East Asian)	Inheritance	SIFT	PolyPhen-2	Mutation taster	SASA value (Å^2^)	ΔΔ*G* (kcal/mol)	ACMG scoring	ACMG pathogenicity
p.Tyr7Cys	0.00005 (0.0007)	-	0.00006 (0.00072)	Paternal	Damaging	Probably damaging	Disease causing	165	−0.186	PP3	Uncertain significance
p.Tyr836Cys	0.0001 (0.0013)	-	0.00008 (0.00078)	Paternal	Tolerated	Probably damaging	Disease causing	13.69	−1.441	PP3	Uncertain significance
p.Pro1031His	0.0005 (0.0076)	0.0009 (0.005)	0.00051 (0.0071)	Paternal	Damaging	Probably damaging	Disease causing	87	−0.314	PS2^a^ + PP1 + PP3	Likely Pathogenic ^b^
Homozygous p.Pro1031His	-	-	-	AR	-	-	-	-	-	-	-
p.Gly1545Ser	-	-	0.00001 (0)	Paternal	Damaging	Probably damaging	Disease causing	3.42	-1.04	PP1 + PP3	Uncertain significance
p.Val151Serfs^∗^27	-	-	-	Paternal	-	-	-	-	-	PVS1 + PM2	Likely pathogenic
p.Arg239^∗^	-	-	-	*De novo*	-	-	-	-	-	PVS1 + PM2 + PS2	Pathogenic
p.Arg838^∗^	-	-	-	Paternal	-	-	-	-	-	PVS1 + PM2	Likely pathogenic
p.Ser1601_Ter1604del_ext133	-	-	-	*De novo*	-	-	-	-	-	PVS1 + PM2 + PS2	Pathogenic

*Abbreviations: ACMG, Standards and guidelines for the interpretation of sequence variants by the American College of Medical Genetics and Genomics; AR, autosomal recessive; ExAC, Exome Aggregation Consortium; KG, 1000 Genomes database; PM2, absent in population databases; PS2, *de novo* (paternity and maternity confirmed); PP1, cosegregation with disease in multiple affected family members; PP3, multiple lines of computational evidence support a deleterious effect on the gene/gene product; PVS1, predicted null variant in a gene where loss of function (LOF) is a known mechanism of disease; SASA, solvent-accessible surface area; SIFT, Sorting Intolerant From Tolerant; ΔΔ*G*, protein stability indicated by free energy change. ^*a*^*De novo* missense variant was reported. ^*b*^Heterozygous p.Pro1031His could be considered as variant of undetermined significance (VUS) owing to its relatively high frequency in East Asian population.*

The molecular effects of the missense variants were further predicted by protein modeling using I-TASSER. As shown in Figure 2C, Tyr7 and Pro1031 are located in protein surface, with the SASA values of 165 and 87 Å^2^, respectively. In contrast, Tyr836 and Gly1545 are deeper in protein cores, with the SASA value of 13.69 and 3.42 Å^2^, respectively. In the wild type of DEPDC5 monomer, Tyr7 residue is located in a loop region with no hydrogen bond to near residues. On the contrary, p.Tyr7Cys formed a new bond with residue Asn672 and would have mild influence on protein folding (the ΔΔ*G* was −0.186 kcal/mol). Under normal conditions, Tyr836 residue forms two hydrogen bonds with Asp253 and Gln820. Conversely, p.Tyr836Cys destroyed the hydrogen bond with Gln820, which resulted in an alteration from short β sheet to loop structure. The difference of biochemical property of the amino acids, as the replacement of a ring-contained aromatic amino acid tyrosine by an aliphatic amino acid cysteine, would have strong influence on protein folding and affect the protein stability (ΔΔ*G* of −1.441 kcal/mol). Normally, the Pro1031 residue is located in the loop region. When proline was replaced by histidine (p.Pro1031His), a new bond with Arg997 was formed and would lead to decreased structural stability (ΔΔ*G* of −0.314 kcal/mol). Gly1545 is located in a loop region close to β sheet with no hydrogen bond to near residues. Three new hydrogen bonds generated when the Gly1545 is replaced by Ser1545, which decreases the structural stability (ΔΔ*G* of −1.04 kcal/mol). These data suggested that the four missense mutations changed the hydrogen bonds and potentially affected the protein stability.

### Phenotype of *DEPDC5* Variants

**FIGURE 3:**
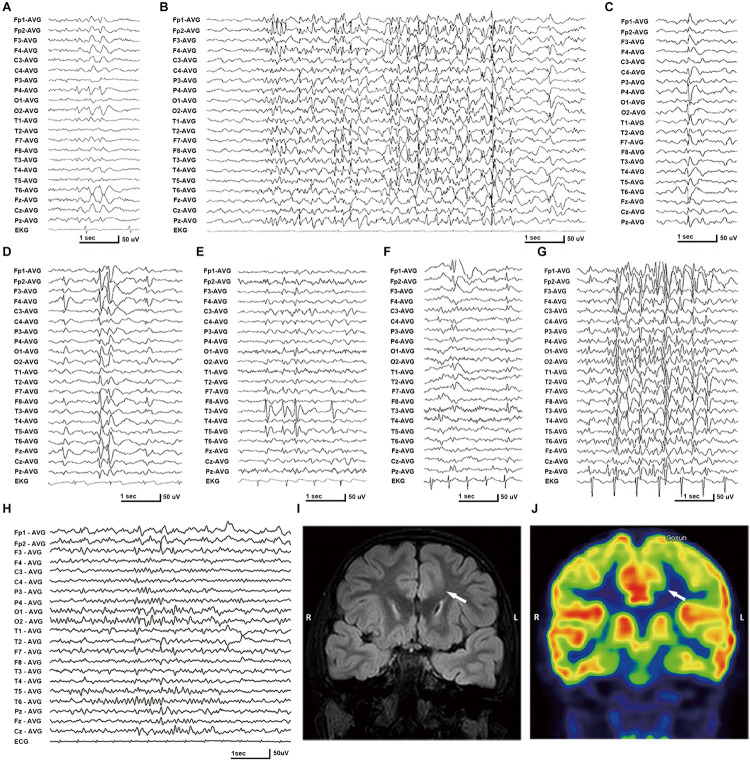
Electroencephalography (EEG) and imaging data of the patients with *DEPDC5* mutations. **(A)** EEG of proband in family A (II-1) shows spikes and waves predominantly in the right parietal region. **(B,D)** Proband in family C (II-1) shows generalized polyspikes and slow waves **(B)** and multifocal discharges **(C,D)**. **(E)** Proband in family E (II-1) shows spikes or spikes and waves in the left temporal lobe. **(F,G)** Proband in family H (II-1) shows both focal spikes and waves or spikes in the left frontal or temporal region **(F)** and generalized discharges **(G)**. **(H)** The case with homozygous p.Pro1031His in family K (II-2) shows spikes or slow waves predominantly in the left frontal lobe; MRI shows focal cortical dysplasia in the anterior cingulate region of frontal lobe **(I)**, which shows hypometabolic signal in PET **(J)**.

In this cohort, eight *DEPDC5* mutations were identified in 12 unrelated families featured by focal epilepsies. All probands had focally originated seizures or focal discharges on EEG recordings. Eight of the probands and another affected individual were diagnosed as focal (partial) epilepsy with FS +. Four of their parents had FS. Other phenotypes included rolandic epilepsy, TLE, unclassified focal epilepsy, and FLE with FCD (homozygous mutation). All cases with heterozygous mutations, including the cases with truncating mutations, presented mild phenotype with good responses to antiepileptic therapy. The case with homozygous mutation and MCD also became seizure free after 1.5 years of frequent seizures. The clinical information of patients with *DEPDC5* mutations is summarized in Table 1, and their representative EEG and imaging data are shown in Figures 3A–J.

The patient carrying the truncating mutation p.Val151Serfs^∗^27 (Figure 1, Family A: II-1) had a single FS at the age of 4 years. She presented with episodic vertigo since the age of 9, which usually lasted for 30–60 s without precipitating factor. Interictal EEG showed spikes and waves predominantly in the right parietal region (Figure 3A). A diagnosis of simple sensory seizure was considered. The attacks occurred at about three times per day and were controlled by levetiracetam monotherapy.

The patient with the truncating mutation p.Arg239^∗^ (Figure 1, Family B: II-1) had first febrile seizure at the age of 15 years. It was generalized tonic–clonic seizures (GTCSs), which lasted for 3–5 min and occurred four times per year during fever. Interictal EEGs showed sharp waves or spikes and slow waves predominantly in the left temporal area. The seizures were controlled by levetiracetam monotherapy.

The proband of the family with p.Arg838^∗^ had focal epilepsy (Figure 1, Family C: II-1). She was a 31-year-old woman who had her first seizure at 7 years of age. She presented with seizures that started with blank staring, automatism, and then limb jerks, lasting 30–90 s, one of which was recorded as a complex partial seizure during EEG monitoring. Interictal EEGs revealed generalized polyspikes and slow waves (Figure 3B), as well as multifocal discharges that tended to be generalized (Figures 3C,D). Her seizures responded well to valproate. Her younger brother (Figure 1, Family C: II-2) had occasional secondarily GTCS (sGTCS).

The patient with termination codon mutation p.Ser1601_Ter1604del_ext133 (Figure 1, Family D: II-1) had both febrile seizures and afebrile seizures that were complex partial seizures and occurred three to five times per year. Interictal EEGs recorded focal-sharp slow-wave discharges predominantly in the frontal and central areas. Seizure was controlled by levetiracetam monotherapy.

The affected individual with p.Tyr7Cys (Figure 1, Family E: II-1) had TLE with left hippocampal atrophy and discharges in the left temporal lobe on EEGs (Figure 3E), which was consistent with the phenotypic characteristics of the case reported previously (Tsai et al., 2017). He became seizure free after the application of oxcarbazepine.

In the family with p.Tyr836Cys, the two affected family members (Figure 1, Family F: II-4, III-1) had epilepsy with antecedent FS. The seizure was sGTCS. Interictal EEGs showed bilateral spike-and-wave discharges predominantly in the frontal area.

In this cohort, heterozygous p.Pro1031His was identified in four unrelated families with eight individuals involved, including a pair of monozygotic twins (Figure 1, Family G: II-1, II-2) with rolandic epilepsy and three families with FEFS + /FS (Figure 1, Family H to J, Table 1). An affected individual with FEFS + (Figure 1, Family H: II-1) showed both focal and generalized discharges on EEGs (Figures 3F,G). The girl with homozygous p.Pro1031His (Figure 1, Family K: II-2) had seizures since she was 3 years old, which usually started with a feeling of fear and followed by loss of consciousness that lasted for around 10 s. She had frequent seizures up to eight times per day for 1.5 years and became seizure free in recently for 1 year with a combination of levetiracetam (50 mg/kg/day), oxcarbazepine (15 mg/kg/day), and clonazepam (1 mg/night). EEG and neuroimaging confirmed the diagnosis of FLE with FCD (Figures 3H–J). One of the heterozygous p.Pro1031His mutations was from her mother, and the other one possibly originated *de novo*. Her paternity was confirmed by alignment of the segregated sequence variants, and all other variants detected were either maternal or paternal. p.Pro1031His has also been reported previously as a *de novo* mutation (Carvill et al., 2015).

The proband of the family with p.Gly1545Ser mutation (Figure 1, Family L: II-1) had two febrile seizures (sGTCS) at the age of 3 and 7 years. His brain imaging and EEG were normal.

### Genotype–Phenotype Correlation

In the present study, six *DEPDC5* mutations were identified in eight unrelated familial cases with FEFS + /FS. These mutations included one termination codon mutation (*de novo*), two truncating mutations (one of *de novo*), and three missense mutations (10 individuals who cosegregated with the mutations in each family). Previously, three *DEPDC5* mutations associated with FS and one mutation associated with FEFS + have been reported (Martin et al., 2014; Pippucci et al., 2015; Ricos et al., 2016; Baldassari et al., 2019). These findings suggested that *DEPDC5* variants were potentially associated with FEFS + /FS. On the other hand, homozygous p.Pro1031His was detected in a case with FLE and FCD. *DEPDC5* variants have been reported to be associated with MCD (including FCD), and a second hit caused by mosaic somatic mutations was suggested (Ribierre et al., 2018). To explore the association between *DEPDC5* variants and MCD or FS/FS +, we systematically reviewed all *DEPDC5* mutations (Supplementary Table S2).

To date, 123 *DEPDC5* mutations, including 87 null mutations and 35 missense mutations, two insertion–deletion mutations, have been identified in 170 unrelated cases. Most of the cases were featured by focal epilepsies (96.4%), except six cases with unclassified epilepsy. Thirty-nine cases were associated with MCD (22.9%). Among the cases without MCD, 113 cases presented as focal epilepsy and 12 cases (nine families and three *de novo*) presented as FEFS + /FS.

**FIGURE 4:**
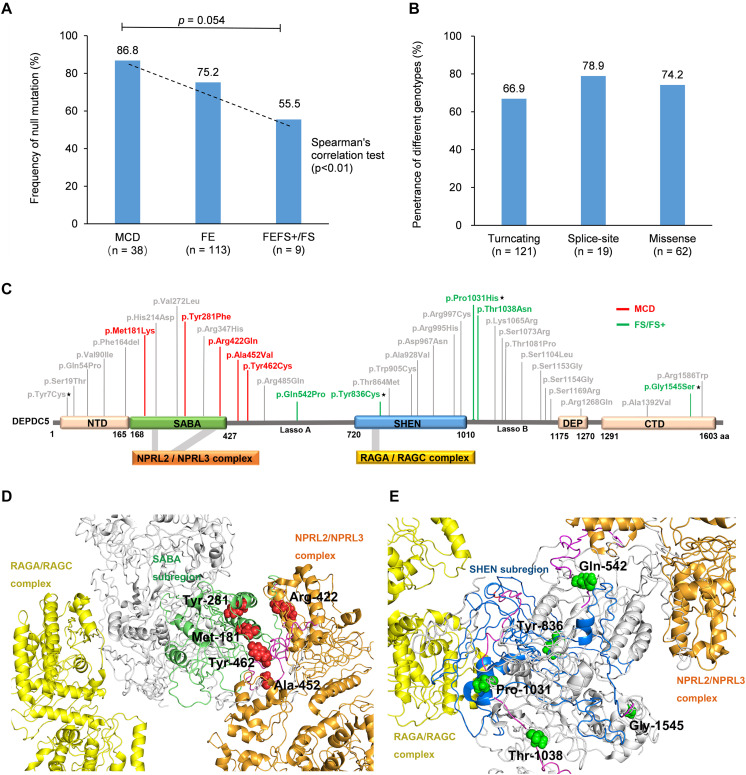
Genotype–phenotype correlations of *DEPDC5* mutations. **(A)** The frequency of null mutations in *DEPDC5* for each phenotype. The values are expressed as the percentage of cases with null mutations (cases with null mutations/total cases) in each group. **(B)** The penetrance of different genotypes of *DEPDC5* mutations. Chi-square test or Fisher’s exact test was used for statistical analysis of the differences. Spearman’s correlation test was used for correlation analysis. **(C)** Schematic diagram of DEPDC5 domain and position of the missense mutations. Malformation of cortical development (MCD) and focal epilepsy with febrile seizures plus/febrile seizures (FEFS+/FS) associated mutations were colored red and green, respectively. The mutations reported in this study are indicated by black stars; the others were from literature. **(D,E)** The spatial location of MCD and FEFS+/FS associated mutations in 3D protein structure. The colors indicated in **D,E** for domains are the same as those in **(C)**.

When we compared the mutation type of different groups, it was found that MCD group had a tendency of higher frequency of null mutation than the patients with FEFS + /FS (excluding p.Pro1031His, Figure 4A). Considering that the patients with MCD commonly presented frequent and refractory seizures (Baulac et al., 2015), a correlation between phenotype severity and null mutation was suggested. *DEPDC5* mutations were originally reported in familial focal epilepsies, but asymptomatic carriers were common. We therefore compared the penetrance of different genotypes. The familial cases with truncating mutations had a penetrance of 66.9%. No statistical significance in penetrance was found among the families with truncating mutations, splice-site mutations, and missense mutations (Figure 4B). The overall penetrance of *DEPDC5* mutations was 70.3% (142/202).

We further mapped the locations of the heterozygous missense variants on DEPDC5 (Figure 4C). The variants distributed over all DEPDC5. However, all MCD-associated heterozygous missense variants clustered around SABA domain and were close to the binding sites to NPRL2/NPRL3 complex (Figure 4D). MCD-related missense mutation identified in this study (p.Pro1031His) was not included for sub-regional analysis owing to its homozygous nature. In contrast, the FEFS + /FS-associated variants were located around SHEN domain and a distance away from NPRL2/NPRL3 complex or RAGA/RAGC complex (Figure 4E). The other three SABA domain-located missense mutations were associated with focal epilepsies (c.640C > G/p.His214Asp and c.814G > T/p.Val272Leu) or SUDEP (c.1040G > A/p.Arg347His), and whether these mutations were associated with FCD was unknown owing to the lack of neuroimaging data (Supplementary Table S2). The other five mutations in SHEN domain were excluded from the association with FCD by neuroimaging examinations (Supplementary Table S2). The three-dimensional protein modeling demonstrated more clearly the location of mutations in DEPDC5 (Supplementary Figures S2a,b^4^).

We analyzed evidence from five clinical-genetic aspects that potentially disclose the association between *DEPDC5* variants and MCD or FEFS + /FS. Evidence from all five clinical-genetic aspects suggested MCD as a phenotype of *DEPDC5* variants; and evidence from four aspects and one possible evidence from sub-regional implication aspect suggested FEFS + /FS as a phenotype of *DEPDC5* variants (Table 3).

**TABLE 3:** Five-dimensional evaluation of phenotypes of *DEPDC5* variants.

	MCD	FEFS + /FS
**1. Repetition:** variants recurrently identified in unrelated cases of homogenous phenotype, or significantly high frequency or hotspot in patients	**Yes.** Identified in 39 unrelated cases from different cohorts	**Yes.** Nine unrelated cases from different reports.
**2. Genotype–phenotype correlation:** for heterogeneous phenotypes, a phenotype was within the spectrum that is correlated with genotype	**Yes.** Homozygous mutation and second-hit brain mosaic mutations, possible high frequency of null mutations	**Yes.** Mainly missense mutations
**3. Inheritance pattern:** cosegregation in families with AD/AR inheritance, or *de novo* origination	**Yes.** Mainly in families with AD inheritance, *de novo* in five cases	**Yes.** Mainly in families with AD inheritance, *de novo* in three cases
**4. Genetic quantitative correlation:** correlation between genetic impairment and phenotype severity	**Yes.** Tendency of high frequency of null mutations, or bi-allelic mutations (including brain mosaicism) in severe phenotype	**Yes.** The mild phenotype with mainly missense mutations that were potentially less functionally impaired
**5. Molecular sub-regional implications:** sub-regional or sub-molecular effects of genetic variants, or distinct functional alteration/mechanism	**Possible.** Heterozygous missense variants clustered in SABA domain and were close to the binding sites to NPRL2/NPRL3 complex	**Possible.** Heterozygous missense variants located away from NPRL2/NPRL3 complex or RAGA/RAGC complex

*Abbreviations: MCD, malformation of cortical development; FEFS + /FS, focal epilepsy with febrile seizures plus/febrile seizures; AD, autosomal dominant; AR, autosomal recessive; SABA, structural axis for binding arrangement.*

## Discussion

In the present study, we identified eight *DEPDC5* mutations in 12 unrelated families from a cohort of 305 patients affected by focal epilepsy (3.9%), including homozygous mutation in a case with FCD. In contrast, no *DEPDC5* mutation was identified in the 91 patients with generalized epilepsies. Thirteen of the 19 affected individuals (68.4%) in this study had FEFS + /FS, suggesting a potential role of *DEPDC5* mutations in FEFS + /FS. Our further analysis revealed potential genotype–phenotype correlations and sub-regional implications of *DEPDC5* variants, which would help understanding the mechanism underlying phenotypical variation.

*DEPDC5* is located on chromosome 22q12.3 and encodes a ubiquitous protein that inhibits the mTOR pathway (Bar-Peled et al., 2013; Marsan and Baulac, 2018). Homozygous *Depdc5^–/–^* embryos of rats died *in utero* owing to global growth delay. In contrast, heterozygous *Depdc5*^±^ rats had altered cortical neuron excitability and firing patterns but without developmental abnormalities or spontaneous electro-clinical seizures (Marsan et al., 2016). These findings suggested a potential quantitative correlation between genetic impairment and phenotype severity and that heterozygous mutations would potentially cause mild phenotype or susceptibility alterations. All cases with heterozygous mutations in this study presented mild phenotype with good responses to antiepileptic therapy, and most individuals have become seizure free. Heterozygous mutations also presented an overall penetrance of 70.3%, which was lower than that in genes of high pathogenic potential like *SCN1A* (Meng et al., 2015). These findings suggested that heterozygous *DEPDC5* mutations were less pathogenic, coincident with the evidence from heterozygous knockout.

In this study, 13 individuals in eight families with *DEPDC5* mutations had FEFS + /FS. Previously, four FS-related families with *DEPDC5* mutations, including two truncating mutations and two missense mutations, have been reported (Supplementary Table S2) (Martin et al., 2014; Pippucci et al., 2015; Ricos et al., 2016; Baldassari et al., 2019). Mutations in 11 of the 12 families inherited in a dominant pattern or originated *de novo*. Further analysis revealed that FEFS + /FS had a lower frequency of null mutation than MCD or other focal epilepsies; and missense mutations associated with FEFS + /FS were located away from the binding sites to NPRL2/NPRL3 or RAGA/RAGC. To define FEFS + /FS as a phenotype of *DEPDC5* variants, we tried to evaluate evidence from five clinical-genetic aspects. Evidence from four aspects, including repetition, genotype–phenotype correlation, inheritance pattern, and genetic quantitative correlation, suggested FEFS + /FS as a phenotype of *DEPDC5* variants. FEFS + /FS associated heterozygous missense variants located away from NPRL2/NPRL3 complex or RAGA/RAGC complex, which was a possible evidence in molecular sub-regional implication aspect (Table 3).

Previous studies have showed that *DEPDC5* mutations are associated with diverse focal epileptic phenotypes, ranging from mild rolandic epilepsy to severe MCD-associated epilepsies (Baulac, 2016). Mechanisms underlying the phenotypic variation were unclear, especially for the severe phenotype like MCD. Previously, brain somatic *DEPDC5* mutations, in addition to the germline mutations, have been identified in two patients with MCD, suggesting a second-hit mechanism (Baulac et al., 2015; Ribierre et al., 2018). The present study identified a homozygous *DEPDC5* mutation (p.Pro1031His) that was associated with FCD. To our knowledge, this was the first report on homozygous *DEPDC5* mutation in patients with FCD, which provided direct evidence on association between bi-allelic *DEPDC5* mutation and MCD. Our further analysis revealed that MCD was more frequently associated with null mutations (Figure 4A); and MCD-associated heterozygous missense mutations were located on SABA domain and were close to the binding sites to NPRL2/NPRL3 complex (Figure 4D), provided additional possible explanations for the association between *DEPDC5* mutations and MCD. DEPDC5 exerts inhibitory effect on mTOR pathway through binding with NPRL2/NPRL3 complex (Shen et al., 2018). It is therefore possible that mutations closer to the binding site of DEPDC5 to NPRL2/NPRL3 may lead to more severe phenotype like MCD. Evidence from four clinical-genetic aspects, including repetition, genotype–phenotype correlation, inheritance pattern, and genetic quantitative correlation, suggested MCD as a phenotype of *DEPDC5* variants. MCD associated heterozygous missense variants clustered in SABA domain and close to the binding sites of NPRL2/NPRL3 complex, which was a possible evidence in molecular sub-regional implication aspect (Table 3).

In the present study, we identified four deleterious *DEPDC5* mutations that would cause gene haploinsufficiency in four cases. Three of the four cases had FEFS + /FS. Additionally, two of the three missense mutations, excluding p.Pro1031His mutation, had FEFS + /FS. These cases suggest a potential role of *DEPDC5* mutations in FEFS + /FS. However, the pathogenicity of missense mutations warrants further verification, especially on variant p.Pro1031His. Heterozygous p.Pro1031His was identified in four families with mild phenotype, including six individuals with FEFS + /FS that cosegregated with the variant in three small families (Figure 1 and Table 1). Previously, heterozygous p.Pro1031His has been identified as a *de novo* mutation in a patient with late-onset epileptic spasms with focal discharges (Carvill et al., 2015). It could be evaluated to be likely pathogenic by ACMG (Table 2). However, heterozygous p.Pro1031His presents at minor allele fractions (MAFs) of 0.00051 in the general populations and at 0.0071 in East Asian population in gnomAD (Table 2). The pathogenicity of p.Pro1031His could therefore be suspected and was reclassified as likely benign (Baldassari et al., 2019) or variant of undetermined significance. Evaluation of the pathogenicity of a variant is currently challenging (Tang et al., 2019), even for genes with *de novo* mutations (He et al., 2019). Generally, the MAFs are closely related to the prevalence of the phenotypes (Richards et al., 2015), among which mild phenotypes potentially have higher prevalence than severe ones. It was noted that a prevalence of febrile seizures as high as 6.9–8.2% has been reported in East Asian population (Tsuboi and Okada, 1984; Byeon et al., 2018). Our recent study has demonstrated that the damaging effects of variants usually vary and potential present a continuing distribution with overlaps between pathogenic variants and benign variants (Tang et al., 2019). It was therefore possible that heterozygous p.Pro1031His may be less pathogenic and even overlapped with rare variants in general populations. On the other hand, homozygous p.Pro1031His did not present in any populations in gnomAD (Table 2) and was potentially associated with MCD. Previous study showed that homozygous *Depdc5^–/–^* knockout was lethal, whereas heterozygous *Depdc5*^±^ led to subclinical change of neuron excitability (Marsan et al., 2016), suggesting a potential quantitative correlation between genetic impairment and phenotype severity, which would help in understanding the difference in the pathogenicity between homogenous and heterogeneous *DEPDC5* missense variants.

This study has several limitations. Previously, effects of *DEPDC5* variants on protein expressions and interactions have been studied. Mutation Y281F presented slightly decreased protein expression, but the four missense mutations tested, including H214D, Y281F, 542P, and S1154F, did not show impacts on other parameters (Dawson et al., 2020). In a similar study, mutations S442F, A452V, T864M, S1073R, and K1088R presented slightly decreased protein expression; mutations A452V and R485Q led to slight increased activity of target of rapamycin complex 1 (Van Kranenburg et al., 2015). Missense mutations in families with cosegregation have been reported, which provided clinical genetic evidence on the pathogenicity of missense *DEPDC5* variants (Dibbens et al., 2013; Ricos et al., 2016). However, further studies on direct functional impact of *DEPDC5* variants are required, especially for missense variants. The present study suggests that the phenotypical spectrum of *DEPDC5* variants potentially includes FEFS + /FS, which warrants validation on a large cohort of FS-related epilepsies.

Epilepsy comprises a huge group of heterogeneous phenotypes with heterogeneous genetic etiologies. So far, approximately 1,000 genes have been reported to be associated with epilepsy (Wang et al., 2017). Defining the association between a phenotype and a gene is practical challenging. Previously, we summarized the evidence required to define the associations between epileptic encephalopathies and genes with *de novo* mutations (He et al., 2019). In a more general sense, we now summarized the clinical-genetic aspects that potentially disclose the association between a phenotype and the genetic variants, which is expected to enable the formulation of guideline for defining a phenotype of genetic variants. This is also the first report on the molecular sub-regional effect of *DEPDC5* variants, which may help in evaluating the pathogenicity of *DEPDC5* variants and development of individualized predictive algorithms, as suggested in our recent study (Tang et al., 2019).

## Data Availability Statement

The datasets are included in the Supplementary Material and uploaded to a public database on Figshare, https://figshare.com/s/17679354408cdca72fd7.

## Ethics Statement

The studies involving human participants were reviewed and approved by Ethics Committee of the Second Affiliated Hospital of Guangzhou Medical University. Written informed consent to participate in this study was provided by the participants’ legal guardian/next of kin.

## Author Contributions

YH-Y and WP-L designed and conceptualized the study. LL, Z-RC, H-QX, D-TL, X-RL, PZ, S-ML, BL, NH, and Q-XZ collected and analyzed the data. LL and Z-RC drafted the manuscript for intellectual content. LL, Z-RC, H-QX, D-TL, TS, HM, W-PL, and Y-HY revised the manuscript for intellectual content. H-KL, YM, and D-TL provided software support for 3D structure model. LL and D-TL prepared the figures. All authors have read and approved the final draft of the manuscript.

## Conflict of Interest

The authors declare that the research was conducted in the absence of any commercial or financial relationships that could be construed as a potential conflict of interest.

## Footnotes

1 http://www.rcsb.org

2 http://www.ncbi.nlm.nih.gov/pubmed/

3 http://www.hgmd.cf.ac.uk/ac/index.php

4 https://figshare.com/s/17679354408cdca72fd7
