# Supplementary material for: DEPDC5 Variants Associated Malformations of Cortical Development and Focal Epilepsy With Febrile Seizure Plus/Febrile Seizures: The Role of Molecular Sub-Regional Effect
Source: Front Neurosci. 2020 Aug 11;14:821. doi: 10.3389/fnins.2020.00821 (PMC7432260; doi:10.3389/fnins.2020.00821)
Supplement: Supplementary file 1 [file Table_1.DOC]

**Table S1** Panel gene list (483 genes)

| *ABAT* | *ABCC2* | *ABCC8* | *ACADM* | *ACO2* | *ACOX1* | *ACP1* | *ACSF3* | *ACTA2* | *ACTB* |
| --- | --- | --- | --- | --- | --- | --- | --- | --- | --- |
| *ACY1* | *ADAR* | *ADCK3* | *ADK* | *ADSL* | *AFG3L2* | *AGTR2* | *ALDH4A1* | *ALDH5A1* | *ALDH7A1* |
| *ALG13* | *AMACR* | *AMT* | *ANK3* | *ANKRD11* | *AP4E1* | *ARG1* | *ARHGEF15* | *ARHGEF9* | *ARSA* |
| *ARX* | *ASAH1* | *ASIC1* | *ASPA* | *ASPM* | *ASS1* | *ATIC* | *ATN1* | *ATP1A2* | *ATP1A3* |
| *ATP2A2* | *ATP6AP2* | *ATP6V0A2* | *ATP7A* | *AUH* | *BCKDK* | *BDNF* | *BRAT1* | *BRD2* | *BTD* |
| *C10ORF2* | *CA1* | *CACNA1A* | *CACNA1E* | *CACNA1G* | *CACNA1H* | *CACNA2D2* | *CACNG2* | *CALHM1* | *CASK* |
| *CASP9* | *CASR* | *CBS* | *CCDC88C* | *CCL3* | *CCL4* | *CCM2* | *CDK5* | *CDKL5* | *CHD2* |
| *CHRFAM7A* | *CHRNA2* | *CHRNA4* | *CHRNA5* | *CHRNA7* | *CHRNB2* | *CLCN2* | *CLCN4* | *CLCNKB* | *CLEC18A* |
| *CLIC2* | *CLN3* | *CLN5* | *CLN6* | *CLN8* | *CNP* | *CNR1* | *CNTF* | *CNTN2* | *CNTNAP2* |
| *COG6* | *COG8* | *COL4A1* | *COQ2* | *COQ9* | *CPA6* | *CPS1* | *CPT2* | *CSTB* | *CTDP1* |
| *CTSD* | *CUL4B* | *CYP4F11* | *D2HGDH* | *DCX* | *DDC* | *DEPDC5* | *DGKD* | *DHFR* | *DIAPH3* |
| *DLX2* | *DLX5* | *DNM1* | *DPM1* | *DPYS* | *DYRK1A* | *EFHC1* | *EFHC2* | *EGF* | *EHMT1* |
| *EIF2AK3* | *ELOVL4* | *ELP4* | *EMX2* | *EN2* | *EPM2A* | *EPM2AIP1* | *FADD* | *FARS2* | *FKTN* |
| *FLNA* | *FOLR1* | *FOS* | *FOXG1* | *FUCA1* | *GABBR1* | *GABBR2* | *GABRA1* | *GABRA2* | *GABRA3* |
| *GABRA4* | *GABRA5* | *GABRA6* | *GABRB1* | *GABRB2* | *GABRB3* | *GABRD* | *GABRE* | *GABRG1* | *GABRG2* |
| *GABRG3* | *GABRP* | *GABRQ* | *GABRR1* | *GABRR2* | *GABRR3* | *GAD1* | *GAMT* | *GATM* | *GBA* |
| *GCDH* | *GCH1* | *GCM2* | *GCSH* | *GJA1* | *GJD2* | *GLB1* | *GLDC* | *GLRA1* | *GLRB* |
| *GLUD1* | *GM2A* | *GOSR2* | *GPHN* | *GPR56* | *GPR98* | *GRIA1* | *GRIA2* | *GRIA3* | *GRIA4* |
| *GRIK1* | *GRIK2* | *GRIN1* | *GRIN2A* | *GRIN2B* | *GRIN2C* | *GRIN2D* | *GRIN3A* | *GRIN3B* | *GRM1* |
| *GRN* | *HAX1* | *HCCS* | *HCFC1* | *HCN1* | *HCN2* | *HCN4* | *HDAC4* | *HEPACAM* | *HEXA* |
| *HEXB* | *HLCS* | *HNRNPU* | *HOXA1* | *HPD* | *HSD17B10* | *HTR1A* | *IDH2* | *IDS* | *IER3IP1* |
| *IL6ST* | *IMPA2* | *INA* | *IQSEC2* | *JRK* | *JUN* | *KARS* | *KCNA1* | *KCNA2* | *KCNA6* |
| *KCNAB1* | *KCNAB2* | *KCNAB3* | *KCNB1* | *KCNC1* | *KCNC2* | *KCNC4* | *KCND1* | *KCND2* | *KCNE1L* |
| *KCNE2* | *KCNF1* | *KCNG1* | *KCNG4* | *KCNH2* | *KCNH3* | *KCNH8* | *KCNJ1* | *KCNJ10* | *KCNJ11* |
| *KCNJ2* | *KCNJ3* | *KCNJ5* | *KCNJ6* | *KCNJ9* | *KCNK1* | *KCNK3* | *KCNK7* | *KCNK9* | *KCNMA1* |
| *KCNMB2* | *KCNMB3* | *KCNMB4* | *KCNN2* | *KCNQ1* | *KCNQ2* | *KCNQ3* | *KCNQ4* | *KCNQ5* | *KCNS2* |
| *KCNS3* | *KCNT1* | *KCNV1* | *KCNV2* | *KCTD7* | *KDM5C* | *KLK1* | *KRIT1* | *L1CAM* | *LAMA2* |
| *LAMB1* | *LBR* | *LGI1* | *LGI2* | *LGI4* | *LIAS* | *LIFR* | *LMBRD1* | *MANBA* | *MAOB* |
| *MAP2* | *MBD5* | *MBTPS2* | *ME2* | *MECP2* | *MED17* | *MEF2C* | *MFSD8* | *MIB1* | *MMADHC* |
| *MOCS1* | *MOCS2* | *MOG* | *MSN* | *MTHFR* | *MTMR9* | *MTR* | *NAGA* | *NDE1* | *NDP* |
| *NDUFV1* | *NF1* | *NGLY1* | *NHLRC1* | *NIPA2* | *NPRL2* | *NPRL3* | *NPY* | *NRXN1* | *NSDHL* |
| *NSF* | *NTRK1* | *NTRK2* | *OPA1* | *OPHN1* | *OPRM1* | *OTX2* | *PAFAH1B1* | *PAH* | *PALLD* |
| *PARK2* | *PC* | *PCDH19* | *PDCD10* | *PDHA1* | *PDHX* | *PDYN* | *PEX1* | *PEX10* | *PEX12* |
| *PEX13* | *PEX14* | *PEX16* | *PEX19* | *PEX2* | *PEX26* | *PEX3* | *PEX5* | *PEX6* | *PFKL* |
| *PFKM* | *PHF6* | *PHGDH* | *PHOX2A* | *PIGA* | *PIGL* | *PIGN* | *PIGO* | *PIGV* | *PIR* |
| *PKHD1* | *PLCB1* | *PNKD* | *PNKP* | *PNPO* | *POLG* | *POLG2* | *PPP1R3C* | *PPT1* | *PRF1* |
| *PRICKLE1* | *PRICKLE2* | *PRIMA1* | *PRNP* | *PRODH* | *PRRT2* | *PSAP* | *PSAT1* | *PSEN1* | *PTEN* |
| *PTH* | *PTS* | *QDPR* | *RAB39B* | *RANBP2* | *RBFOX1* | *RBPJ* | *RELN* | *RHAG* | *RHOA* |
| *RMND1* | *RNASEH2A* | *RNF213* | *ROGDI* | *RPS6KA3* | *SCARB2* | *SCN1A* | *SCN1B* | *SCN2A* | *SCN2B* |
| *SCN3A* | *SCN3B* | *SCN4A* | *SCN5A* | *SCN7A* | *SCN8A* | *SCN9A* | *SDHA* | *SEPSECS* | *SERPINI1* |
| *SEZ6* | *SHANK3* | *SLC12A1* | *SLC12A5* | *SLC12A6* | *SLC16A1* | *SLC17A5* | *SLC19A3* | *SLC1A1* | *SLC1A2* |
| *SLC1A3* | *SLC20A2* | *SLC25A12* | *SLC25A13* | *SLC25A22* | *SLC2A1* | *SLC35A2* | *SLC35A3* | *SLC35C1* | *SLC46A1* |
| *SLC4A3* | *SLC52A2* | *SLC6A11* | *SLC6A13* | *SLC6A19* | *SLC6A8* | *SLC9A3* | *SLC9A6* | *SLC9A9* | *SMARCA2* |
| *SMARCA4* | *SNIP1* | *SOBP* | *SPAST* | *SPR* | *SPTAN1* | *SRGAP2* | *SRPX2* | *ST3GAL3* | *ST3GAL5* |
| *STRADA* | *STXBP1* | *SUOX* | *SV2A* | *SYN1* | *SYNGAP1* | *SYP* | *SYT11* | *SZT2* | *TBC1D24* |
| *TBCE* | *TBX1* | *TCF4* | *TICAM1* | *TK2* | *TLR3* | *TMEM67* | *TNF* | *TPP1* | *TPRXL* |
| *TREX1* | *TRMT44* | *TRPM6* | *TSC1* | *TSC2* | *TSEN2* | *TSEN34* | *TSEN54* | *TSPO* | *TUBA1A* |
| *TUBA8* | *TUBB2B* | *TUBGCP6* | *U2AF1* | *UBC* | *UBE2A* | *UBE3A* | *VAMP2* | *VLDLR* | *VPS13A* |
| *WARS* | *WDR45* | *ZEB2* |  |  |  |  |  |  |  |
