# Supplementary material for: DEPDC5 Variants Associated Malformations of Cortical Development and Focal Epilepsy With Febrile Seizure Plus/Febrile Seizures: The Role of Molecular Sub-Regional Effect
Source: Front Neurosci. 2020 Aug 11;14:821. doi: 10.3389/fnins.2020.00821 (PMC7432260; doi:10.3389/fnins.2020.00821)
Supplement: Supplementary file 2 [file Table_2.DOC]

**Table S2 Epilepsy-related *DEPDC5* mutations and their phenotypes (each line represents an unrelated case/family)**

| **Nucleotide change** | **Amino acid change** | **Inheritance** | **Phenotypes (details and number of affected individuals)** | | | **Ref** |
| --- | --- | --- | --- | --- | --- | --- |
| **Cases of focal epilepsy with MCD** | | | | | | |
| c.21C>G | p.Tyr7* | Familial | BOSD, FFEVF (BOSD with FLE 1; FLE 2, PLE 1, TLE 5, ASD 1, unaffected 3) | | | 1,2 |
| c.128_129insC | p.Asn45Glnfs*3 | Unknown | Hemimegalencephaly (Hemimegalencephaly 1) | | | 10 |
| c.279+1G>A | - | Familial | FCD, FFEVF (FCD with FLE 1; F-TLE 1) | | | 1,2 |
| c.279+1G>A | - | Familial | FCD IIa, SHE (FCD+ SHE with DR 1; unclassified 1) | | | 3 |
| c.418C>T | p.Gln140* | Familial | BOSD (BOSD with FLE 2; FLE 3, FE 1, unaffected 4) | | | 1 |
| c.484-1G>A | - | Familial | FCD IIa, NE (FCD with NE 1, NFLE 1, unaffected father mosaic 5% 1) | | | 4 |
| c.542T>A | p.Met181Lys | Familial | FCD, IS, FE (IS at onset, FCD+FE with DR later 1, father with FS) | | | 3 |
| c.624+1G>A | - | Unknown | FCD IIb (FCD 1) | | | 10 |
| c.715C>T | p.Arg239* | Familial ^a^ | FCD, FLE (FLE+ FCD with DR 1, unaffected father 1) | | | 3 |
| c.715C>T | p.Arg239*+  p.Arg422* (somatic) | Familial | FCD, FFEVF (FCD with FFEVF 1, plus brain somatic mutation c.1264C>T /p.Arg422*; FCD with FFEVF 2; FE 4; unaffected 5) | | | 4,5 |
| c.783_786delTGAG | p.Asn261Lysfs*11 | Unknown | FCD IIb (FCD 1) | | | 10 |
| c.842A>T | p.Tyr281Phe | Familial | FCD, IS, FE (FCD with IS 1; multifocal 1, unclassified 1) | | | 6 |
| c.856C>T | p.Arg286* +  p.Gln289* (somatic) | Familial ^a^ | FCD IIa, NFLE (FCD with NFLE 1, plus brain somatic mutation c.865C>T/p.Gln289* in seizure-onset zone; unaffected mother 1) | | | 11 |
| c.856C>T | p.Arg286* | Familial ^a^ | FCD IIa, FLE (FCD+FLE with DR 1, unaffected mother 1) | | | 3 |
| c.982C>T | p.Arg328* | De novo | FCD, IS, FE (FC+ IS+ FE with DR 1) | | | 3 |
| c.1165dupC | p.Arg389Profs*2 | Unknown | FCD, SHE (FCD with SHE 1) | | | 3 |
| c.1218-18_1218-15  delTGTT (VUS) | - | Unknown | FCD IIb (FCD 1) | | | 10 |
| c.1264C>T | p.Arg422* | Unknown | FCD, NFLE (FCD with NFLE 1) | | | 4 |
| c.1264C>T | p.Arg422* | Familial | FCD IIa, SHE (FCD+ SHE with DR 1; FE1; unclassified 1) | | | 3 |
| c.1265G>A (VUS) | p.Arg422Gln | Familial ^a^ | Hemimegalencephaly (Hemimegalencephaly 1, unaffected mother 1) | | | 10 |
| c.1310delA | p.Asn437Metfs*21 | Familial ^a^ | FCD Ic, FE (FCD+ FE with DR 1, unaffected mother 1） | | | 3 |
| c.1355C>T (VUS) | p.Ala452Val | Unknown | FCD IIb (FCD 1) | | | 10 |
| c.1385A>G | p.Tyr462Cys | Unknown | hMCD, FLE (hMCD with FLE 1) | | | 3 |
| c.1400_1401insGG | p.Phe467Leufs*51 | Familiar ^a^ | FCD llb, FE (FCD with FE 1, unaffected father 1) | | | 3 |
| c.1663C>T | p.Arg555* | Familial | FCD IIa (FCD 2, FE 2, unaffected 1) | | | 7 |
| c.1663C>T | p.Arg555* | Familial | FCD, SHE (FCD with SHE 1; unclassified 5, unaffected 1) | | | 3 |
| c.1759C>T | p.Arg587* | Familial | FCD (FCD with FE 2) | | | 4 |
| c.2390delA | p.Gln797Argfs*18 | De novo | FCD, IS (FCD with IS 1) | | | 6 |
| c.3019C>T (c.3046C>T) | p.Gln1007*  (p.Gln1016*) | Unknown | FCD, SUDEP (FCD 1) | | | 8 |
| c.3021+1G>A | - | Familiar ^a^ | FCD I, FE (FCD+ FE with DR 1, unaffected father 1) | | | 3 |
| c.3092C>A | p.Pro1031His  (homozygous) | Familial ^a^ | FLE with MCD (one allele inherited from unaffected mother and one allele originated de novo mutation) | | | This study |
| c.3696+5G>A | - | De novo | Polymicrogyria, FE (Polymicrogyria with FE 1) | | | 12 |
| c.3994C>T | p.Arg1332* | Familial ^a^ | FCD, TLE (FCD with TLE 1, unaffected father 1) | | | 12 |
| c.3994C>T | p.Arg1332* | Familial ^a^ | FCD, SUDEP (FCD with SUDEP 1, unaffected father 1) | | | 8 |
| c.4031T>A | p.Leu1344* | Familial ^a^ | FCD, SHE (FCD+SHE with DR 1, unaffected father 1) | | | 3 |
| c.4260delG | p.Glu1421Argfs*153 | Unknown | FCD, FE (FCD with FE 1) | | | 9 |
| c.4460delC  (c.4187delC) | p.Ala1487ValfsX78  (p.Ala1396Valfs*78) | De novo | Hemimegalencephaly (Hemimegalencephaly 1) | | | 13 |
| c.4662_4663delAG | p.Asp1556* | De novo | Pachygyria (Pachygyria 1) | | | 14 |
| c.4674G>A | p.Trp1558* | Unknown | FCD, FE (FCD+ FE with DR 1) | | | 3 |
| **Cases of focal epilepsy without MCD** | | | | | | |
| c.20A>G (VUS) | p.Tyr7Cys | Unknown | TLE (TLE 1) | | | 22 |
| c.20A>G (VUS) | p.Tyr7Cys | Familial ^a^ | TLE (TLE 1, unaffected father 1) | | | This study |
| c.59-1G>C | - | Familial | RE (RE 2, unaffected 2) | | | 15 |
| c.161A>C | p.Gln54Pro | Familial ^a^ | TLE (TLE 1, unaffected mother 1) | | | 12 |
| c.193+1G>A | - | Familial | FFEVF, IS (FLE 1, IS 1, IS with OLE 1, unclassified 1, unaffected 1) | | | 2,6 |
| c.232delC | p.Arg78Glyfs*2 | Familial | FE, IS (FF with IS 1; FE 1) | | | 3 |
| c.232delC | p.Arg78Glyfs*2 | Unknown | SHE (SHE with DR 1) | | | 3 |
| c.268G>A (VUS) | p.Val90Ile | Familial ^a^ | RE (RE 1, unaffected father 1) | | | 15 |
| c.299delT | p.Val100Glyfs*3 | Unknown | SHE (SHE with DR 1) | | | 3 |
| c.319C>T | p.Gln107* | Familial ^a^ | SHE (SHE with DR 1, unaffected father 1) | | | 3 |
| c.378delC | p.Tyr127Ilefs*51 | Familial ^a^ | SHE (SHE with DR 1, unaffected mother 1) | | | 3 |
| c.418C>T | p.Gln140* | Familial | OLE, TLE (OLE 1, TLE 1, unaffected 1) | | | 16 |
| c.422_423insCTGG | p.Gly142Trpfs*3 | Familial ^a^ | IS, FE (IS with multifocal spike and waves 1, asymptomatic father, multiple other unaffected carriers) | | | 3 |
| c.435G>A | p.Trp145* | Unknown | FE (FE 1) | | | 3 |
| c.435G>A | p.Trp145* | Familial | NFLE (NFLE 2, unaffected 1) | | | 12 |
| c.454_455delAT | p.Met152Valfs*6 | Unknown | FE (FE 1) | | | 9 |
| c.488_490delTGT | p.Phe164del | Familial | FFEVF (FLE 2, F-TLE 8, NFLE 4, OLE 1, TLE 9, unclassified 11) | | | 2 |
| c.492_496delTCGTT | p.Arg165Tyrfs*14 | Familial ^a^ | NFLE (NFLE 1, unaffected mother 1) | | | 12 |
| c.526C>T | p.Gln176* | Familial ^a^ | NFLE (NFLE 1, unaffected 2) | | | 12 |
| c.640C>G | p.His214Asp | Familial ^a^ | NFLE (NFLE 1, unaffected father 1) | | | 12 |
| c.727C>T | p.Arg243* | Unknown | FE, GE (FE+GE with DR 1) | | | 3 |
| c.727C>T | p.Arg243* | Unknown | FE (FE 1) | | | 15 |
| c.727C>T | p.Arg243* | Unknown | SHE (SHE with DR 1) | | | 3 |
| c.727C>T | p.Arg243* | Familial ^a^ | NFLE (NFLE 1, unaffected son 1) | | | 12 |
| c.730C>T  (c.646C>T) | p.Gln244*  (p.Gln216*) | Familial | FE (FE 6) | | | 17 |
| c.790delA | p.Arg264Glufs*9 | Unknown | FE (FE with DR 1) | | | 3 |
| c.814G>T (VUS) | p.Val272Leu | Familial | RE (RE 2, unaffected 1) | | | 15 |
| c.918C>G | p.Tyr306* | Familial | FTLE (FTLE 2) | | | 18 |
| c.982C>T | p.Arg328* | Familial | FTLE (FTLE 4) | | | 5 |
| c.985delA | p.Thr329Leufs*7 | Familial | NFLE, TLE (NFLE 4, TLE 1, unclassified 1, unconfirmed seizure 1, unaffected 4) | | | 12 |
| c.943_944insG | p.Asn315Argfs*4 | Familial | FE, FLE (FE 4, FLE1, unaffected 1) | | | 3 |
| c.1092_1099ins  GGATTTGG | p.Val367Glyfs*40 | Familial | FLE (FLE 2, unaffected 1) | | | 12 |
| c.1114C>T | p.Gln372* | Familial | FFEVF (FE 2) | | | 5 |
| c.1114C>T | p.Gln372* | Unknown | FLE (FLE with DR 1) | | | 3 |
| c.1122delA | p.Leu374Phefs*30 | Familial | FFEVF (FE 7, unaffected 3) | | | 5 |
| c.1264C>T | p.Arg422* | Familial | FLE (FLE 2, unaffected 1) | | | 12 |
| c.1310delA | p.Asn437Metfs*21 | Familial | FE (FE with DR 1; FE 1) | | | 3 |
| c.1355C>T | p.Aal452Val | Familial | FFEVF (TLE 2) | | | 2 |
| c.1393C>T | p.Gln465* | Familial | FE (FE 4) | | | 9 |
| c.1454G>A | p.Arg485Gln | Familial | FTLE (FTLE 2) | | | 5 |
| c.1459C>T | p.Arg487* | Familial | FFEVF (NFLE 2, unclassified 1, unaffected 1) | | | 2 |
| c.1459C>T | p.Arg487* | Familial | NFLE (NFLE 2, unaffected 1) | | | 19 |
| c.1474C>T | p.Arg492* | Unknown | IS, FE (IS at onset, then FE with DR1) | | | 3 |
| c.1474C>T | p.Arg492* | Unknown | FLE (FLE 1) | | | 3 |
| c.1546delG | p.Val516* | Unknown | FE (FE 1) | | | 23 |
| c.1555C>T | p.Gln519* | Familial | FLE, IS (FLE 1, IS 1) | | | 6 |
| c.1663C>T | p.Arg555* | Familial | FFEVF (FLE 4, F-TLE 3, NFLE 2, TLE 2, multifocal 1, unclassified 2, unaffected 10) | | | 2 |
| c.1663C>T | p.Arg555* | Familial | FE (FE with DR 2, FE 1) | | | 3 |
| c.1750_1756del  CATGCTG | p.Leu584Phefs*12 | Familial | FE (FE 2) | | | 9 |
| c.1845delG | p.Arg615Serfs*47 | Familial ^a^ | SHE (SHE with DR 1, unaffected mother 1) | | | 3 |
| c.1909C>T | p.Arg637* | Unknown | NFLE (NFLE 1) | | | 12 |
| c.2355-2A>G | - | Familial | NFLE (NFLE 2) | | | 19 |
| c.2512C>T | p.Arg838* | Familial | FE (FE 1, unclassified 1, unaffected 1) | | | This study |
| c.2512C>T | p.Arg838* | Familial | SHE, FLE (SHE 1; FLE 1) | | | 3 |
| c.2512C>T | p.Arg838* | Familial ^a^ | SHE, FE (SHE with FE 1, unaffected mother 1) | | | 3 |
| c.2512C>T | p.Arg838* | Unknown | SHE (SHE with DR 1) | | | 3 |
| c.2527C>T | p.Arg843* | Unknown | SUDEP, TLE (TLE with SUDEP 1) | | | 8 |
| c.2527C>T | p.Arg843* | Familial | FFEVF (FLE 1, F-TLE 1, NFLE 1, unclassified 1) | | | 2 |
| c.2527C>T | p.Arg843* | Familial ^a^ | FFEVF (TLE 1, unaffected father 1) | | | 20 |
| c.2527C>T | p.Arg843* | Familial | FFEVF (FE 1, multifocal 1, unaffected 1) | | | 20 |
| c.2527C>T | p.Arg843* | Unknown | FLE (FLE 1; NA for others) | | | 3 |
| c.2527C>T | p.Arg843* | Familial | FE, SHE (SHE with DR 1; FE 1) | | | 3 |
| c.2591C>T | p.Thr864Met | Unknown | C-TLE (C-TLE 1) | | | 20 |
| c.2593C>T | p.Arg865* | Familial | FE (FE 3, unaffected 1) | | | 15 |
| c.2620C>T | p.Arg874* | Familial ^a^ | FE, SHE (FE with SHE 1, unaffected mother 1) | | | 3 |
| c.2620C>T | p.Arg874* | Unknown | FLE (FLE 1) | | | 3 |
| c.2715G>T | p.Trp905Cys | Familial ^a^ | FE (FE with DR 1, unaffected father 1) | | | 3 |
| c.2760C>A | p.Tyr920* | Familial ^a^ | FLE (FLE 1, unaffected mother 1) | | | 3 |
| c.2783C>T | p.Ala928Val | Familial ^a^ | FE (FE 1, unaffected father 1) | | | 3 |
| c.2847_2862delGCCTGTGTCACCGCCA | p.Ala951Profs*38 | Familial ^a^ | FE, SHE (FE+SHE with DR 1, unaffected father 1) | | | 3 |
| c.2899G>A | p.Asp967Asn | Familial ^a^ | RE (RE 1, unaffected father 1) | | | 22 |
| c.2984G>A | p.Arg995His | Unknown | NFE (NFE 1) | | | 22 |
| c.2989C>T | p.Arg997Cys | Familial ^a^ | FE (FE with DR 1, unaffected father 1) | | | 3 |
| c.3092C>A | p.Pro1031His | De novo | ES (late-onset with focal discharge ES 1) | | | 6 |
| c.3092C>A | p.Pro1031His | Familial | RE (RE 2, unaffected father 1) | | | This study |
| c.3194A>G (VUS) | p.Lys1065Arg | Unknown | FE (FE 1, NA for others) | | | 9 |
| c.3217A>C | p.Ser1073Arg | Unknown | FFEVF (TLE 1) | | | 2 |
| c.3241A>C | p.Thr1081Pro | Familial ^a^ | ECSWS (ECSWS 1, unaffected mother 1) | | | 12 |
| c.3259C>T | p.Arg1087* | Familial | NFLE (NFLE 2, unaffected father 1) | | | 19 |
| c.3259C>T | p.Arg1087* | Familial | FE (FE 2) | | | 3 |
| c.3265-3C>T | - | Familial | TLE (TLE 2, unaffected father 1) | | | 12 |
| c.3311C>T | p.Ser1104Leu | Familial | FFEVF (TLE 3) | | | 2 |
| c.3330+5G>C | - | Familial ^a^ | TLE (TLE1, unaffected father 1) | | | 3 |
| c.3417delA | p.Ile1139Metfs*24 | Familial ^a^ | RE (RE 1, unaffected father 1) | | | 15 |
|  | p.Ser1153Gly | Familial ^a^ | RE (RE 1, unaffected father 1) | | | 15 |
| c.3461C>T | p.Ser1154Phe | Familial | TLE (TLE 1, unclassified 1, unaffected 1) | | | 12 |
| c.3507C>G | p.Ser1169Arg | Familial ^a^ | FE (FE 1, unaffected mother 1) | | | 3 |
| c. 3563+4A>G | - | Unknown | FLE (FLE with DR 1) | | | 3 |
| c.3631_3642delinsATACACCTCCAT | Val1211_Leu1214delinsIleHisLeuHis | Familial ^a^ | FE, SHE (FE+ SHE with DR 1, unaffected mother 1) | | | 3 |
| c.3696+5G>A | - | Unknown | FE (FE with DR 1) | | | 3 |
| c.3802C>T | p.Arg1268* | De novo | FFEVF (TLE 1, unaffected 1) | | | 2 |
| c.3803G>A | p.Arg1268Gln | Familial ^a^ | TLE (TLE 1, unaffected father 1) | | | 12 |
| c.3994C>T | p.Arg1332* | Familial ^a^ | FE, SHE (FE+SHE with DR 1, unaffected mother 1) | | | 3 |
| c.4033+5A>G | - | Familial ^a^ | TLE (TLE 1, unaffected mother 1) | | | 12 |
| c.4107G>A | p.Trp1369* | Familial | FFEVF (FLE 1, NFLE 2, TLE 1, unclassified 3, unaffected 6) | | | 2 |
| c.4107G>A | p.Trp1369* | Familial | NFLE (NFLE 3) | | | 19 |
| c.4112delT | p.Leu1371Argfs*14 | Familial | FE, EAF (FE 2, EAF 1) | | | 21 |
| c.4151_4152insC | p.Glu1385* | Familial | SHE, FE (SHE with bifrontal epileptic activity 4) | | | 3 |
| c.4175C>T | p.Ala1392Val | Familial ^a^ | GE (multifocal and generalized epileptic activity 1, unaffected father 1) | | | 3 |
| c.4203+2T>A | - | Familial | FFEVF (FLE 3, TLE 1, unclassified 1) | | | 22 |
| c.4397G>A | p.Trp1466* | Familial | FFEVF (FLE 1, unclassified 4, unaffected 1) | | | 2 |
| c.4397G>A | p.Trp1466* | Familial | FFEVF (FLE 2, unaffected 1) | | | 2 |
| c.4420delG | p.Glu1474Lysfs*100 | Familial | FE, SHE (FE+SHE with DR 2, unaffected 1) | | | 3 |
| c.4520-2A>G | - | Unknown | FE (FE 1) | | | 3 |
| c.4567C>T | p.Gln1523* | Unknown | NFLE (NFLE 1) | | | 5 |
| c.4606C>T | p.Gln1536* | Familial | FFEVF (FLE 5, unaffected 5) | | | 2 |
| c.4756C>T (VUS) | p.Arg1586Trp | Unknown | TLE (TLE 1, unaffected 1) | | | 22 |
| ex.1_8del | - | Familial | FE (FE 1, unclassified 1) | | | 3 |
| ex.2_3del | - | Unknown | FE (FE with DR 1; unclassified 1) | | | 3 |
| ex.8_19del | - | Familial ^a^ | FE, SHE (FE with SHE 1, unaffected father 1) | | | 3 |
| ex.28_38del | - | Unknown | FE (FE 1) | | | 24 |
| ex. 31_42del | - | Unknown | FE (FE 1) | | | 3 |
| ex. 40del | - | Unknown | FE (FE1; unclassified 2) | | | 3 |
| **Cases of focal epilepsy with febrile seizures plus/ febrile seizures (FEFS+/FS)** | | | | | | |
| c.715C>T | p.Arg239* | De novo | FEFS+ (FEFS+1) | | | This study |
| c.918C>G | p.Tyr306* | Familial | FS, FE, EAF (FS+ EAF 1, FS+ FE 1) | | | 21 |
| c.1625A>C | p.Gln542Pro | Familial | FEFS+, FE (NE 1, FS+ OLE 1, T-OLE 1, unaffected 1) | | | 12 |
| c.2507A>G | p.Tyr836Cys | Familial | FEFS+ (FEFS+ 2, unaffected 1) | | | This study |
| c.2527C>T | p.Arg843* | Familial | FS, FFEVF (C-TLE 1, FLE 2, FS 1, F-TLE 1, P-OLE 1, unaffected 5) | | | 20 |
| c.3092C>A | p.Pro1031His | Familial | FEFS+ (FS 1, FEFS+ 1) | | | This study |
| c.3092C>A | p.Pro1031His | Familial | FEFS+ (FS 1, FEFS+ 1) | | | This study |
| c.3092C>A | p.Pro1031His | Familial | FEFS+ (FS 1, FEFS+ 1) | | | This study |
| c.3113C>A | p.Thr1038Asn | De novo | FS (FS 1) | | | 3 |
| c.450delG | p.Val151Serfs*27 | Familial ^a^ | FEFS+ (FEFS+ 1, unaffected father 1) | | | This study |
| c.4633G>A | p.Gly1545Ser | Familial | FEFS+ (FS 1, FEFS+ 1) | | | This study |
| c.4802_*40delGTGCCCCGTGAGGCCAGGCTGCACCTGTGCTGGGGGAAGGTGGGTGAGCCA | p.Ser1601_Ter1604del_ext133 | De novo | FEFS+ (FEFS+ 1) | | | This study |
| **Cases of other epilepsy** | |  | |  |  |  |
| c.56G>C | p.Ser19Thr | Unknown | SUDEP (SUDEP 1, NA for others) | | | 8 |
| c.856C>T | p.Arg286* | Unknown | SUDEP, NE (NE with SUDEP 1, NA for others) | | | 8 |
| c.1040G>A | p.Arg347His | Unknown | SUDEP (SUDEP 1, NA for others) | | | 8 |
| c.2620C>T | p.Arg874* | Familial ^a^ | SHE (Interictal EEG normal, SHE with DR 1; unaffected father with somnambulism 1) | | | 3 |
| c.3230_3234delCCATG | p.Ala1077Aspfs*82 | Familial ^a^ | GE (GE 1; unaffected mother 1) | | | 3 |
| c.3802C>T | p.Arg1268* | Unknown | IS (EEG hypsarrhythmia, IS with DR 1) | | | 3 |

Abbreviations: ASD, autism spectrum disorder; BOSD, bottom-of-the-sulcus dysplasia; C-TLE, centro-temporal lobe epilepsy; DR, drug resistance; EAF, epilepsy with auditory features; ECSWS, epilepsy with continuous spike-and-waves during slow-wave sleep; ES, epileptic spasms; FCD, focal cortical dysplasia; FE, focal epilepsy; FFEVF, familial focal epilepsy with variable foci; FLE, frontal lobe epilepsy; FS, febrile seizure; FS+, febrile seizure plus; FTLE, familial temporal lobe epilepsy; F-TLE, fronto-temporal lobe epilepsy; GE, generalized epilepsy; hMCD: hemispheric malformation of cortical development; IS, infantile spasms; MCD, malformations of cortical development; NA, not available; NE, nocturnal epilepsy; NFE, nocturnal focal epilepsy; NFLE, nocturnal frontal lobe epilepsy; OLE, occipital lobe epilepsy; P-OLE, parieto-occipital lobe epilepsy; RE, rolandic epilepsy; SHE: Sleep-related hypermotor epilepsy; SUDEP, sudden unexpected death in epilepsy; TLE, temporal lobe epilepsy; T-OLE, temporal-occipital lobe epilepsy; VUS, variant of unknown significance in original report.

^a^ only one affected individual as sporadic cases. The mutation was inherited from their unaffected mother or father.

Nucleotide and amino acid numbering are according to *DEPDC5* reference transcript NM 001242896.1 (reference protein NP 001229825.1), with +1 corresponding to the A of the ATG translation initiation codon in the reference sequence. Variants using different transcripts in the original reports are indicated in parenthesis.

**References:**

1. Scheffer, I.E. et al. Mutations in mammalian target of rapamycin regulator DEPDC5 cause focal epilepsy with brain malformations. *Annals of Neurology* **75**, 782-787 (2014).

2.  Dibbens, L.M. et al. Mutations in DEPDC5 cause Familial focal epilepsy with variable foci. *Nature Genetics* **45**, 546-551 (2013).

3.  Baldassari, S. et al. The landscape of epilepsy-related GATOR1 variants. *Genet Med* (2018).

4.  Baulac, S. et al. Familial focal epilepsy with focal cortical dysplasia due to DEPDC5 mutations. *Annals of Neurology* **77**, 675-683 (2015).

5. Ishida, S. et al. Mutations of DEPDC5 cause autosomal dominant focal epilepsies. *Nature Genetics* **45**, 552-555 (2013).

6.  Carvill, G.L. et al. Epileptic spasms are a feature of DEPDC5 mTORopathy. *Neurology Genetics* **1**, e17 (2015).

7. Scerri, T. et al. Familial cortical dysplasia type IIA caused by a germline mutation in DEPDC5. *Annals of Clinical and Translational Neurology* **2**, 575-580 (2015).

8. Bagnall, R.D. et al. Exome-based analysis of cardiac arrhythmia, respiratory control, and epilepsy genes in sudden unexpected death in epilepsy. *Ann Neurol* **79**, 522-34 (2016).

9.  Weckhuysen, S. et al. Involvement of GATOR complex genes in Familial focal epilepsies and focal cortical dysplasia. *Epilepsia* **57**, 994-1003 (2016).

10. D'Gama, A.M. et al. Mammalian target of rapamycin pathway mutations cause hemimegalencephaly and focal cortical dysplasia. *Ann Neurol* **77**, 720-5 (2015).

11. Ribierre, T. et al. Second-hit mosaic mutation in mTORC1 repressor DEPDC5 causes focal cortical dysplasia–associated epilepsy. *Journal of Clinical Investigation* **128**, 2452-2458 (2018).

12. Ricos, M.G. et al. Mutations in the mammalian target of rapamycin pathway regulators NPRL2 and NPRL3 cause focal epilepsy. *Ann Neurol* **79**, 120-31 (2016).

13. Mirzaa, G.M. et al. Association of MTOR Mutations With Developmental Brain Disorders, Including Megalencephaly, Focal Cortical Dysplasia, and Pigmentary Mosaicism. *JAMA Neurology* **73**, 836 (2016).

14. Cen, Z.G.Y.L. De novo mutation in DEPDC5 associated with unilateral pachygyria and intractable epilepsy. *Seizure* **50**, 1 - 3 (2017).

15. Lal, D. et al. DEPDC5 mutations in genetic focal epilepsies of childhood. *Ann Neurol* **75**, 788-92 (2014).

16.  Perucca, P. et al. Real-world utility of whole exome sequencing with targeted gene analysis for focal epilepsy. *Epilepsy Research* **131**, 1-8 (2017).

17. Nascimento, F.A., Borlot, F., Cossette, P., Minassian, B.A. & Andrade, D.M. Two definite cases of sudden unexpected death in epilepsy in a family with a DEPDC5 mutation. *Neurology Genetics* **1**, e28 (2015).

18.  Striano, P. et al. DEPDC5 mutations are not a frequent cause of Familial temporal lobe epilepsy. *Epilepsia* **56**, e168-e171 (2015).

19.  Picard, F. et al. DEPDC5 mutations in families presenting as autosomal dominant nocturnal frontal lobe epilepsy. *Neurology* **82**, 2101-2106 (2014).

20. Martin, C. et al. A recurrent mutation in DEPDC5 predisposes to focal epilepsies in the French-Canadian population. *Clin Genet* **86**, 570-4 (2014).

21.  Pippucci, T. et al. Epilepsy with auditory features: A heterogeneous clinico-molecular disease. *Neurol Genet* **1**, e5 (2015).

22.  Tsai, M.H. et al. DEPDC5 mutations in Familial and sporadic focal epilepsy. *Clin Genet* **92**, 397-404 (2017).

23. Tsai, M. et al. Molecular Genetic Characterization of Patients With Focal Epilepsy Using a Customized Targeted Resequencing Gene Panel. *Frontiers in Neurology* **9**(2018).

24. Bonaglia, M.C. et al. Partial deletion of DEPDC5 in a child with focal epilepsy. *Epilepsia Open* **1**, 140-144 (2016).
