# Supplementary figures and images for: DEPDC5 Variants Associated Malformations of Cortical Development and Focal Epilepsy With Febrile Seizure Plus/Febrile Seizures: The Role of Molecular Sub-Regional Effect
Source: Front Neurosci. 2020 Aug 11;14:821. doi: 10.3389/fnins.2020.00821 (PMC7432260; doi:10.3389/fnins.2020.00821)

**Figure S1** Sanger sequencing for each family

**
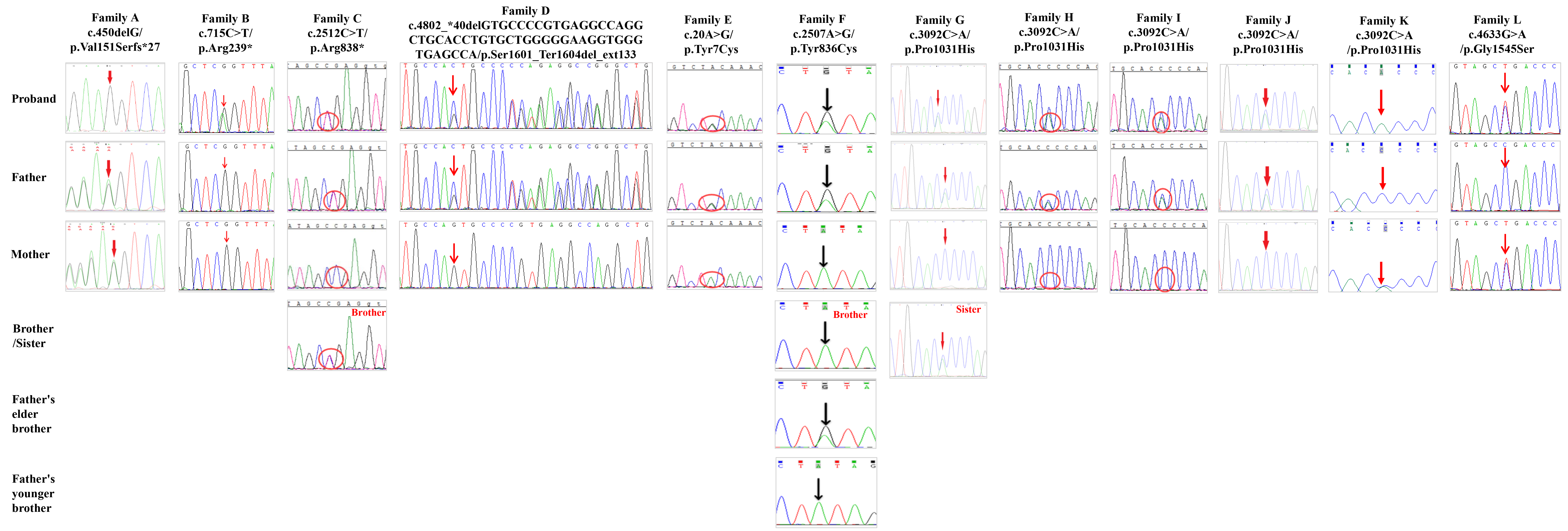
**

Supplement: Supplementary file 3 [file Data_Sheet_1.doc]
